# Split-inducing indels in phylogenomic analysis

**DOI:** 10.1186/s13015-018-0130-7

**Published:** 2018-07-16

**Authors:** Alexander Donath, Peter F. Stadler

**Affiliations:** 1 Center for Molecular Biodiversity Research (zmb), Zoological Research Museum Alexander Koenig (ZFMK), Adenauerallee 160, 53113 Bonn, Germany; 2 Bioinformatics Group, Department of Computer Science, Interdisciplinary Center for Bioinformatics, Universität Leipzig, Härtelstraße 16–18, 04107 Leipzig, Germany; 3 Competence Center for Scalable Data Services and Solutions Dresden/Leipzig, German Centre for Integrative Biodiversity Research (iDiv), and Leipzig Research Center for Civilization Diseases, Universität Leipzig, Härtelstraße 16–18, 04107 Leipzig, Germany; 4 Max Planck Institute for Mathematics in the Sciences, Inselstraße 22, 04103 Leipzig, Germany; 5 Fraunhofer Institute for Cell Therapy and Immunology, Perlickstraße 1, 04103 Leipzig, Germany; 6 Department of Theoretical Chemistry, University of Vienna, Währinger Straße 17, 1090 Vienna, Austria; 7 Center for non-coding RNA in Technology and Health, Grønegårdsvej 3, 1870 Frederiksberg C, Denmark; 8 Santa Fe Institute, 1399 Hyde Park Rd., Santa Fe, NM87501 USA

**Keywords:** In/del, Splits, Genome-wide multiple sequence alignments, Phylogenomics

## Abstract

**Background:**

Most phylogenetic studies using molecular data treat gaps in multiple sequence alignments as missing data or even completely exclude alignment columns that contain gaps.

**Results:**

Here we show that gap patterns in large-scale, genome-wide alignments are themselves phylogenetically informative and can be used to infer reliable phylogenies provided the gap data are properly filtered to reduce noise introduced by the alignment method. We introduce here the notion of split-inducing indels (*splids*) that define an approximate bipartition of the taxon set. We show both in simulated data and in case studies on real-life data that *splids* can be efficiently extracted from phylogenomic data sets.

**Conclusions:**

Suitably processed gap patterns extracted from genome-wide alignment provide a surprisingly clear phylogenetic signal and an allow the inference of accurate phylogenetic trees.

**Electronic supplementary material:**

The online version of this article (10.1186/s13015-018-0130-7) contains supplementary material, which is available to authorized users.

## Background

Gaps in multiple sequence alignments are usually seen as a nuisance in molecular phylogenetics. In most studies, gaps are treated as missing data or alignment columns with gaps are even removed completely. Indeed, stochastic models of sequence evolution that deal explicitly with insertions and deletions (indels) have been investigated only recently [1, 2]. Detailed evaluation shows an overall improvement of phylogenetic reconstructions when indels are modelled explicitly [3–5]. For instance, the inclusion of insertion and deletion (indel) characters proved useful in the analysis of the phylogeny of the Arctoidea (Mammalia: Carnivora) [6], neognathous birds [7], or fungal families [8]. Nevertheless, there is a negative effect of an increasing density of gap characters in multiple sequence alignments [5]. Furthermore, recent studies have indicated that biases may be introduced when indels are included without precautions in Bayesian and Maximum Likelihood phylogenies [9, 10].

Between these few recent rigorous approaches to include gaps and the dismissal of gaps as missing data, indels have been incorporated in several ways into sequence-based phylogenetic analyses. The simplest one is the coding of gaps as fifth character state. Other authors have suggested the replacement of the gapped regions by a binary matrix that codes presence and/or absence of the respective indel [11]. This binary matrix is then added to the “ungapped” sequence data and employed in tree inference. An extension of this simple indel coding (SIC) approach maximizes the amount of phylogenetic information in a parsimonious way by incorporating all indels [12].

Gaps in alignments are, of course, not features identifiable from the individual sequences. Instead, they appear as derived patterns inferred from sequence comparison only. Nevertheless, they convey a surprising amount of phylogenetic information. Shared multi-residue deletions, for instance, have been used to support hypothesis derived from molecular data in single gene analyses, see e.g. [13]. Multi-residue gaps in nucleotide as well as protein sequences have been reported as useful indicators of monophyletic groups [14]. Single-residue gaps, on the other hand, occur more frequently than multi-residue gaps and show a higher amount of homoplasy, e.g. [15]. The same authors suggest that single-residue gaps should not be removed a priori from a data set based on a large taxon sampling, since they can still contain a phylogenetic signal. Ashkenazy et al. [16] proposed to quantify the reliability of indel characters by measuring the frequency with which they appear in alternative multiple sequence alignments. They show that weighting or filtering indels by reliability in general improves the accuracy of phylogenetic reconstruction.

The few studies of the phylogenetic information content of gap patterns were mostly conducted on limited sets of protein data. Gap patterns are, however, very different between coding and non-coding regions [17]. With the advent of high-throughput sequencing (nearly) complete genomes are becoming available at an increasing pace, from which large-scale genome-wide alignments can be constructed [18, 19]. Phylogenomics capitalizes on these developments and provides a wide diversity of phylogenetic information [20]. We utilize these developments here to address the value of gap patterns from a phylogenomic perspective. Since we aim at using pre-computed genome-wide alignments it is not feasible to evaluate individual gaps by their stability with regard to different alignment methods as proposed in [16]. On the other hand, the size of genome-wide data sets allows us to devise stringent filtering criteria to reduce noise and alignment-specific biases. To this end we focus on the sub-class of indels that define a “reasonably obvious” binary split among the sequences. As gaps are not part of the sequence itself but the result of an alignment algorithm, however, we need to systematically investigate the impact of the alignment method on the phylogenetic information of the gap patterns.

## Theory: inference of split-inducing indels

The encoding of characters from gap patterns is not entirely trivial as soon as indels rather than individual gap characters are to be assessed.

**Fig. 1:**
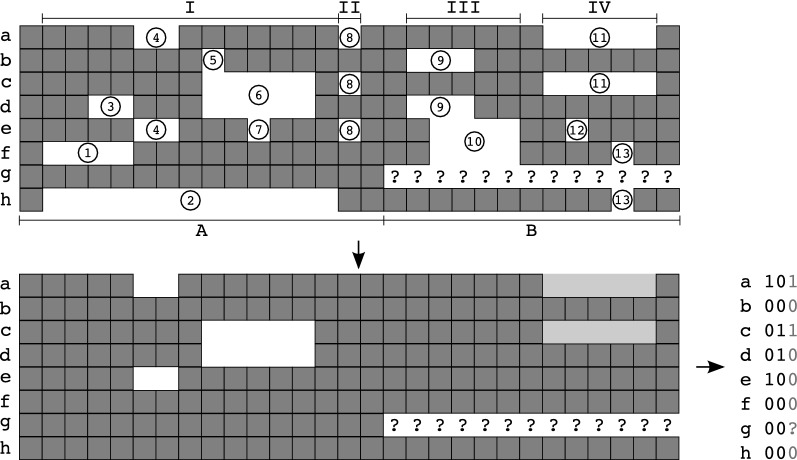
Non-trivial example of the determination of *splids* with size $\ge$ 2 from two concatenated alignments (A and B). Alignment A contains sequence data for all taxa, whereas B lacks sequence information for taxon *g*. At first, all indel loci are determined (I–IV). Second, indel loci are searched for indels constituting *splids*. From locus I only indels (4) and (6) fulfill this criterion. Indels (1) and (3) do not share a common 5’ end. Indel (8) is too short. Indels (9) and (10) of locus III are overlapping *splids*. Whether or not indel (11) is included in the final *splid* set depends on the applied algorithm. In *strict* mode it is not included, due to the single-residue indel (13). In *fuzzy* mode, it is included and taxon *g* is marked as missing data (“?”) in the binary presence/absence coding

We formally define an indel to be a contiguous stretch of gap characters in one or more rows of the alignment. Each indel therefore has a well-defined start and stop column. Its size is defined as the number of consecutive gap characters. Two indels overlap if there is an alignment column that is common to both of them [see, e.g., indel (1) and (3) in Fig. 1]. An indel locus consists of indels that overlap, i.e., a contiguous sequence of alignment columns such that two adjacent columns share at least one indel. By definition, the indels in two indel loci are independent of each other and thus can be treated separately.

We call an indel a *spl*it-inducing *i*n*d*el (*splid*) if it defines an approximate bipartition of the taxon set according to the following rules:

1. Only indels that are present in at least two sequences and have a user-defined minimum size are taken into account. By default, all indels of size at least two are considered. Thus, indels (1), (2), (3), (5), (7), (8), (12), and (13) in Fig. 1 can be ignored.
2. A *splid* cannot overlap another indel that satiesfies the first condition. Thus, indels (9) and (10) are excluded.

*Splids* are coded as binary characters marking their presence/absence pattern in the respective taxon. Missing sequence data in the alignment column of a *splid* is coded as “missing data” (“?”). We optionally filter out *splids* that overlap a single-residue indel occurring in at least two taxa [such as indel (13)]. Applying this “strict mode” removes indel (11), while it is retained in “fuzzy mode”. These alternative treatments of single-position gaps is motivated by the observation that they occur more randomly than multi-residue gaps, while still containing some phylogenetic information [15]. Thus, including these *splids* could increase the number of available characters, although this increases the possibility of conflicting signal.

## Methods

### Implementation

The algorithm for the conversion of alignments to a binary character matrix is implemented in the C++ program gappy. It reads multiple sequence alignments in MAF and FASTA format. The user can select a minimum and maximum indel size for determining *splids*. By default, the output is a FASTA file, containing the binary coded *splid* presence/absence information, and a summary statistic with details about the identified *splids*. Output is also available in PHYLIP and NEXUS format.

### Data sets

#### Simulated data

Indel rates and indel-size distributions are usually estimated based on pairwise alignments (e.g., human-mouse, primates, rodents [21–24]) but differ quite considerably. For example, estimates for the ratio of substitution rates to indel rates between mouse and human are ranging from 8 [24] to 14 [22, 23]. It seems to be a good approximation to apply an indel rate in vertebrates at least as high as between human and mouse, however. Estimates suggest that the frequency of deletions is somewhat higher than the insertion frequency [21, 25, 26], with a ratio of deletion rate $\lambda _d$ to insertion rate $\lambda _i$ ranging from 1.3 to 4. We therefore created three different data sets using the F81 model [27], two indel-size distributions and different indel rates, each consisting of 100 alignments with a length of 100,000 bp (see Additional file 1: Figure S1). The first two data sets use a geometric distribution with similar insertion and deletion rates ($\lambda _{i1} \, =\, \lambda _{i2} \, =\, 0.03106$ and $\lambda _{d1} \,=\, \lambda _{d2} \,=\, 0.04037$) but different probability values ($q_1 \, = \, 0.7$ and $q_2 \, = \, 0.55$, respectively). The third data set follows a Lavalette distribution ($a \,=\, 1.5$, $M \,=\, 120$, $\lambda _{i3} \,=\, 0.02899$, and $\lambda _{d3} \,=\, 0.03768$), which has been suggested as being an appropriate approximation of the indel length distribution in real-life data sets [28, 29]. All data sets were simulated using INDELible V1.03 [29]. The guide tree and background base frequencies were taken from the phastCons17way phastCons tree model file [30] obtained from UCSC^1^ and rescaled to have a maximum root-to-tip distance of 2.

#### ENCODE data

In order to address the problem of how our method behaves under real-life data and genome-scale alignment lengths we created two data sets from the ENCODE [31] project data, based on the December 2007 Multi-Species Sequence Analysis sequence freeze available from UCSC.^2^ The ENCODE data contains sequences of 35 vertebrates orthologous to a representative 1% of the human genome divided among 44 regions. The sequences were aligned with TBA/Multiz [18], a toolkit that has been widely used for whole-genome alignments in large-scale comparative genomics studies [31, 32]. TBA/Multiz produces a set of local alignments (“blocks”) that are stitched together relative to a reference sequence to represent the evolutionary operations, in particular insertions and deletions, that separate the included sequences. The program requires a predefined guide tree that describes the relationship of the species to be aligned. In case of the ENCODE data set this tree is largely based on taxonomic information.

A genome-wide alignment is the result of an extensive similarity search between at least two species. Due to evolutionary changes in genome organization, such as inversions and duplications, two genomes are virtually never completely co-linear, resulting in a decomposition of alignments into syntenic blocks. Practical procedures such as TBA/Multiz also use other features, such as large insertions, missing data in individual species, or low complexity regions, as additional breakpoints, so that relative small alignment blocks are produced. Not all of these blocks contain sequence information from all taxa, both due to missing data in the sequence assemblies and because highly diverged regions of some taxa cannot be reliably recognized as homologous.

The first data set contains only those alignments with sequence information for all 36 organisms. Alignment blocks of two ENCODE regions fulfilled this criteria: ENm001 (498 alignment blocks) and ENm013 (67 alignment blocks). To investigate how the method behaves under a considerable amount of missing data, as it is usually the case for genome wide alignments, we created a second data set, based on all ENCODE alignment regions with sequence information for at least three species.

### Re-alignment without predefined guide trees

The use of a predefined guide tree for the genome alignments could conceivably create a bias in indel positioning. We therefore checked whether such a bias exists and how other commonly used alignment programs perform. To this end we removed all gaps from the ENCODE alignment blocks. The genome-wide alignments thus are used only as a convenient means of extracting orthologous regions.

We applied a similar procedure to the ’true’ alignments of the simulated data set. To mimic the properties of the ENCODE alignments, we first split all simulated alignments in blocks with an average size of 140 bp. After removing all gaps, each block was then re-aligned with a variety of commonly used multiple sequence alignment programs using default settings: ClustalW version 2.0.12 [33], Muscle version 3.7 [34], T-Coffee version 8.97 [35], Prank version 100802 [36], Dialign-TX version 1.0.2 [37], and Mafft version 6.833b [38]. Mafft was run in three different strategies: default mode, L-INS-i, and G-INS-i mode. Dialign-TX differs from all other methods as it creates alignments from local pairwise sequence similarities without the use of explicit gap penalties.

Approximately 2% of the ENCODE regions contain coding exons while the majority covers non-coding sequences, such as introns, UTRs, and intergenic regions. It has been pointed out that, while performing fairly good on these sequences, TBA/Multiz’s results on regions containing non-coding RNAs is not optimal [39]. We therefore additionally tested ProbConsRNA version 1.1 [40], an experimental version of PROBCONS for nucleotide data with parameters estimated from BRAliBASE II via unsupervised training [41].

Following realignment, gaps introduced at the 5′ and 3′ ends of the sequence blocks were considered as artifacts and hence coded as missing data (see also [11]). As individual alignment blocks typically contain sequence information for only a subset of the input taxa, sequences of such missing taxa were also explicitly coded as missing data. Alignment blocks with sequence information for two or more taxa and containing at least one gap character were then concatenated using a custom Perl script (available with the source code of gappy). Note that by construction the delimiting columns of each alignment block do not contain gap characters; concatenation therefore does not affect the gap patterns. From these concatenated alignments we extracted all *splids* $\ge$ 2 bp using gappy in *strict* mode.

### Phylogenetic reconstruction and analysis

#### Model selection and tree reconstruction

Binary model selection was performed using PartitionFinder version 2.1.1 [42] and comparing the BIC scores. Phylogenetic trees were calculated with RAxML version 8.2.11 [43], executing 100 rapid bootstrap inferences and thereafter a thorough ML search. Bootstrap support values were drawn on the best-scoring tree.

#### Tree comparison

Two phylogenetic *n*-taxa trees can be compared using a variety of different distance measures. The most sensitive one is the unweighted Robinson–Foulds (RF) distance ($d_{RF}$) [44], defined as the sum of the number of splits present in exactly one of the two trees. The normalized RF distance ($d'_{RF}$) is then computed by dividing $d_{RF}$ by the maximal possible distance between the two trees, i.e., $d'_{RF} \,=\, d_{RF}/(2n-6)$. The RF measure does not emphasize local similarity, so that trees differing by the placement of a single taxon may have a large RF distance [45]. We therefore also calculated the quartet distance ($d_Q$) [46], defined as the number of quartets that are subtrees of one but not the other input tree, for comparison. The normalized quartet distance, $$d^{\prime}_{Q} {\mkern 1mu} = {\mkern 1mu} {{d_{Q} } \mathord{\left/ {\vphantom {{d_{Q} } {\left( {\begin{array}{*{20}l} n \\ 4 \\ \end{array} } \right)}}} \right. \kern-\nulldelimiterspace} {\left( {\begin{array}{*{20}l} n \\ 4 \\ \end{array} } \right)}}$$, serves as a convenient distance measure between large phylogenetic trees. We use here Phylonet version 3.6.1 [47] and tqDist version 1.0.0 [48] to compare the obtained trees with the respective UCSC guide trees.

## Results

### Simulated alignments

In order to test the phylogenetic signal provided by *splids* we first used simulated sequence data generated with INDELible along a known reference tree. Alignments were computed using nine different methods. PartitionFinder identified the GAMMA model of rate heterogeneity including ascertainment bias correction as the most suitable model for all *splid* alignments. In total 3000 trees were calculated from these alignments and the simulated INDELible reference alignments. On these artificial data set we observe nearly correct trees derived from *splids* (see Additional file 1: Figure S1). On these benign data, the choice of the alignment methods has little effect on the quality of the estimated phylogenies. No RF distances between reconstructed phylogeny and reference tree larger than 4 were observed. This corresponds to a maximum of two splits that are not present in the reference tree. Indeed 84.07% of the trees were identical to the reference tree, and another 15.17% showed an RF distance of 2. Quartet distances draw a similar picture but allow a better differentiation between the results of the respective methods. The overwhelming majority of all trees (97.4%) from all alignment methods have a $d'_{Q} \, \le \, 0.001221$. The tree most dissimilar to the guide tree ($d'_{Q} \,=\, 0.016801$) was calculated based on one of the ClustalW alignments. The alignment program that performed best in terms of similarity to the reference tree was Mafft L-INS-i with an average of $d'_{Q} \,=\, 0.000227$.

### ENCODE genomes

*Data set with sequence information for all taxa.* Depending on the alignment method, the concatenated alignments of the ENCODE data differed quite considerably in length and hence in the total number of gaps. For the small ENCODE data set, ClustalW produced the shortest and Dialign-TX the longest alignment (Table 1). In general, the number of *splids* increased with the number of alignment sites. For the three Mafft algorithms, however, the number of *splids* decreases with increasing alignment length. In particular, Mafft default and Mafft L-INS-i seem to introduce more single-residue gaps or conflicting splits than Mafft G-INS-i.

**Table 1:** Overview of the total number of sites of all alignments per alignment method and the number of derived *splids* with length $\ge$ 2 bp for the ENCODE data set containing only alignments with sequence information for all taxa

Program	Number of sites	Number of *splids*
ClustalW	79,006	793
Dialign-TX	96,990	2163
Mafft	84,105	1021
Mafft L-INS-i	83,578	1245
Mafft G-INS-i	83,123	1279
Muscle	84,577	1378
ProbConsRNA	86,277	1927
Prank	96,622	2047
T-Coffee	84,835	1831
TBA/Multiz	90,726	2032

**Fig. 2:**
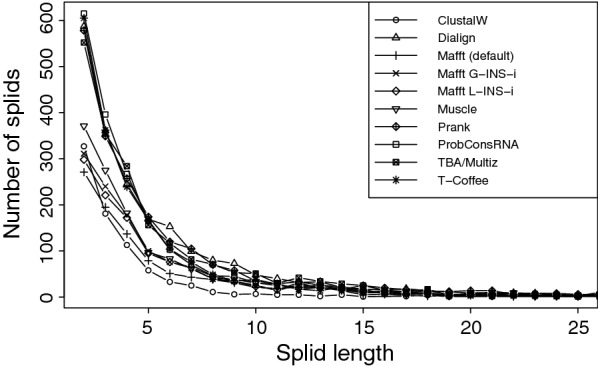
Number of *splids* with a length of $\ge$ 2 bp that have been extracted from the alignments of the ENCODE data set containing sequence information for all taxa

Dialign-TX, T-Coffee, Prank, and ProbConsRNA yield a *splid* length distribution similar to TBA/Multiz (Fig. 2). In comparison, Muscle, ClustalW, and all three Mafft algorithms found considerably fewer shorter *splids*. There is, however, no systematic dependence on design features of the alignment methods such as global versus local alignments or progressive versus consistency based methods.

While the *splid*-based phylogenies are nearly perfect on simulated data, we observed larger deviations that depend at least in part on the alignment methods when applying our approach to real-life data. On the other hand, in real data sets we do not have an absolute ground truth to compare to. Thus, we discuss in following both the quality of the reconstructed phylogenies and the position of interesting taxa in some detail [see Table 2 and machine-readable data online (see Availability)].

The monophyly of Afrotheria and the positioning of tenrec basal to elephant and rock hyrax [49, 50] was always recovered. Only *splid* data derived from the Mafft default alignments placed tenrec basal to armadillo. The position of the placental root is still, at least to some extent, a matter of debate [51–54]. However, results based on the Mafft default alignments and most other alignment programs correctly positioned Afrotheria outside of Boreoeutheria [55]. Only *splid* data obtained from the Muscle, ProbConsRNA, and T-Coffee alignments placed Afrotheria as sister group to Laurasiatheria (ProbConsRNA and T-Coffee) or inside Euarchontoglires (Muscle). The original TBA/Multiz alignments did not contain enough supporting *splids* to position Afrotheria outside of Boreoeutheria, however.

**Table 2:** Detailed comparision of the differences between the ENCODE guide tree and the best maximum likelihood trees calculated from *splid* data derived from various alignment tools

	ClustalW	Dialign-TX	Mafft	Mafft G-INS-i	Mafft L-INS-i	Muscle	Prank	ProbConsRNA	T-Coffee	TBA/Multiz
Afrotheria	×	×	–	×	×	×	×	×	×	×
	Sister group to Boreoeutheria	Sister group to Boreoeutheria		Sister group to Boreoeutheria	Sister group to Boreoeutheria	Within Boreoeutheria	Sister group to Xenarthra	Sister group to (Laurasiatheria, Xenarthra)	Sister group to (Laurasiatheria, Xenarthra)	Sister group to Euarchontoglires
((elephant, rock hyrax), tenrec)	×	×	–	×	×	×	×	×	×	×
Xenarthra (Armadillo)	Sister taxon to Epitheria	Sister taxon to Epitheria	Sister taxon to part of the paraphyletic Epitheria	Sister taxon to Epitheria	Sister taxon to Epitheria	Sister taxon to Epitheria	Sister taxon to Afrotheria	Sister taxon to Laurasiatheria	Sister taxon to Laurasiatheria	Sister group to Epitheria
Boreoeutheria	×	×	×	×	×	–	–	–	–	–
Laurasiatheria	×	×	×	×	×	×	×	×	×	×
Insectivora	×	×	×	×	×	×	×	×	×	×
Chiroptera	×	×	×	×	×	×	×	×	×	×
((rfbat, flying fox), sbbat)	×	×	×	×	×	×	×	–	×	–
Carnivora	×	×	×	×	×	×	×	×	×	×
Horse	(bats, horse)	(Carn., horse)	(Carn., horse)	(Carn., horse)	(cow, horse)	((bats, cow), horse)	((bats, cow), horse)	(((bats, cow), Carn.), horse)	(cow, horse)	(Carn., horse)
Cow	((bats, horse), cow)	(((Carn., horse), bats), cow)	(((Carn., horse), bats), cow)	(bats, cow)	(cow, horse)	(bats, cow)	(bats, cow)	(bats, cow)	(cow, horse)	(((Carn., horse), bats), cow)
Euarchontoglires	×	×	×	×	×	–	×	×	–	×
Glires	×	–	–	–	–	–	×	×	–	×
Rodentia	–	–	–	×	×	–	×	×	–	×
Muroidea	×	×	×	×	×	× Basal within Epitheria	×	×	×	×
Rabbit	Sister taxon to Muroidea	Sister taxon to tree shrew; both sister group to Primata	Basal within Euarchontoglires	Sister taxon to tree shrew; both sister group to Rodentia	Sister taxon to tree shrew; both sister group to Rodentia	Sister taxon to Euarchontoglires	Sister taxon to Rodentia	Sister taxon to Rodentia	Sister taxon to Primata	Sister taxon to Rodentia
Primata	×	×	×	×	×	×	×	–	×	×
Strepsirrhini	×	×	×	×	×	×	×	×	×	×
Platyrrhini	×	×	×	×	×	×	×	×	×	×
(((squirrel monkey, marmoset), owl monkey), dusky titi)	–	–	–	–	–	–	–	–	×	–
Catarrhini	×	×	×	×	×	×	×	×	×	×
Cercopithecidae	×	×	×	×	×	×	×	×	×	×
(((baboon, macaque), vervet), colobus)	–	–	–	×	×	×	×	–	×	–
Hominoidea	×	×	×	×	×	×	×	×	×	×
(((chimp, human), orangutan), gibbon)	×	×	×	×	×	×	–	–	×	–
Tree shrew	In Glires; sister taxon to (Hystricomorpha, Sciuromorpha)	Sister taxon to rabbit; both sister group to Primata	Sister taxon to Rodentia	Sister taxon to rabbit; both sister group to Rodentia	Sister taxon to rabbit; both sister group to Rodentia	Sister taxon to (Hystricomorpha, Sciuromorpha)	Basal within Euarchontoglires	Sister taxon to Strepsirrhini	Sister taxon to remaining (Epitheria, Xenarthra)	Sister taxon to Glires
$d_{RF}$	20	18	18	14	16	22	16	20	20	14
$d'_{RF}$	0.3030	0.2727	0.2727	0.2121	0.2424	0.3333	0.2424	0.3030	0.3030	0.2121
$d_{Q}$	2347	2980	2748	1892	2043	6376	4164	6951	9458	3932
$d'_{Q}$	0.0398	0.0506	0.0467	0.0321	0.0347	0.1082	0.0707	0.1180	0.1606	0.0668

*Splids* (gap length $\ge$ 2 bp) were extracted from the ENCODE regions containing sequence information for all taxa. For each tree the symmetric difference (Robinson–Foulds distance, $d_{RF}$), the normalized RF distance ($d'_{RF}$), the quartet distance ($d_{Q}$, at most 58,905), and the normalized quartet distance ($d'_{Q}$) to the ENCODE guide tree is shown. rfbat = *Rhinolophus ferrumequinum*, sbbat = *Myotis lucifugus*, Carn. = Carnivora. “×” = monophyly/position recovered, “–” = monophyly/position not recovered. See text for details

Three hypotheses concerning the positioning of Xenarthra are discussed in the literature: (1) basal-Afrotheria ((Boreoeutheria, Xenarthra); Exafroplacentalia), e.g. [51, 54], (2) basal-Xenarthra ((Boreoeutheria, Afrotheria); Epitheria), e.g. [56], and (3) basal-Boreoeutheria ((Afrotheria, Xenarthra); Atlantogenata), e.g. [57]. *Splid* data mostly supports the basal-Xenarthra hypothesis. Prank positioned armadillo basal to Afrotheria, whereas ProbConsRNA and T-Coffee placed armadillo basal to Laurasiatheria and therefore inside Boreoeutheria. Interestingly, none of the calculated trees supports the ENCODE guide tree that follows the basal-Afrotheria hypothesis.

**Fig. 3:**
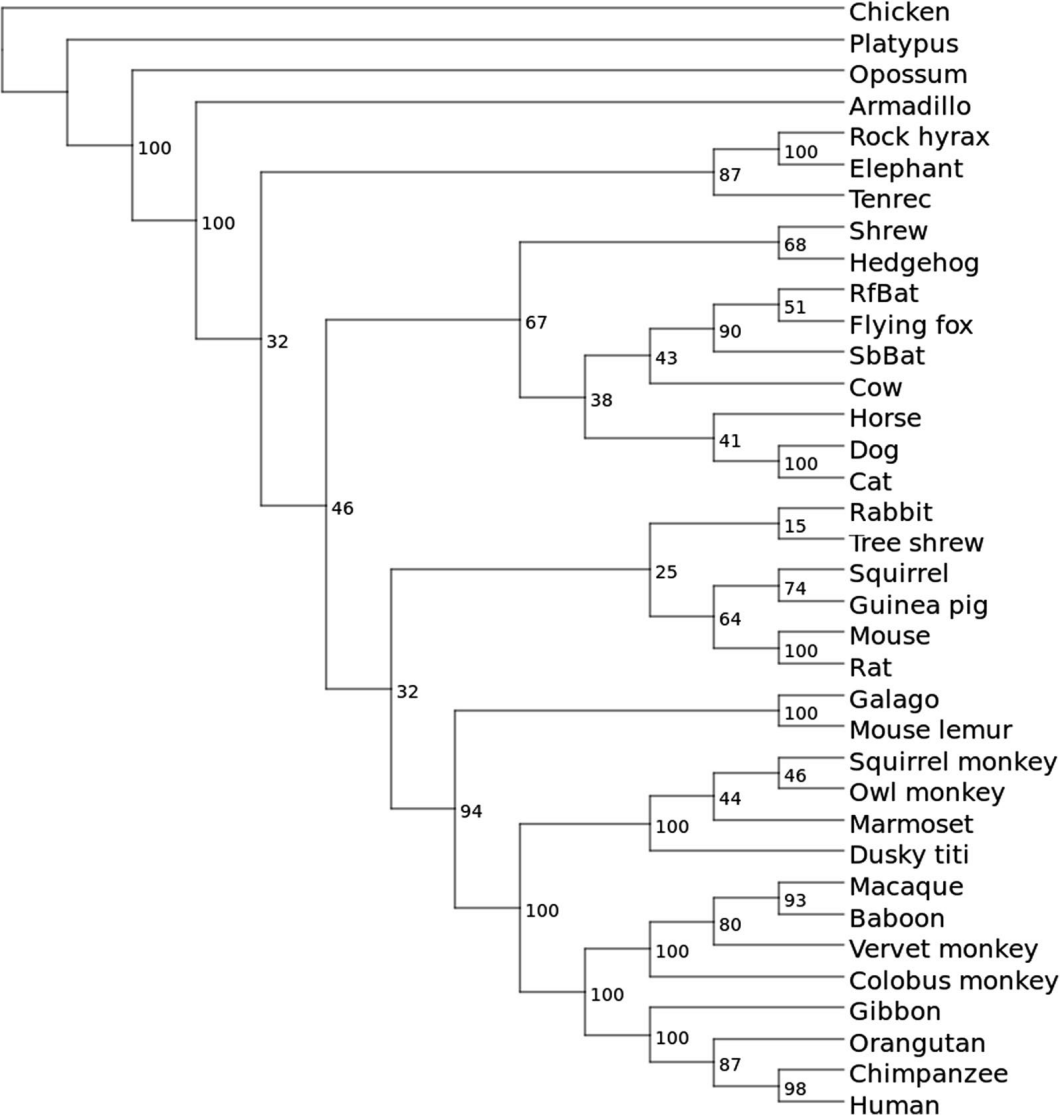
Cladogram with bootstrap values obtained from 100 bootstrap trees calculated by RAxML using *splid* data and the Gamma model with ascertainment bias correction. *Splids* with gap lengths $\ge$2 bp were extracted from the small ENCODE data set that has been re-aligned using Mafft G-INS-i

Monophyly of Laurasiatheria was recovered in all cases. Monophyly was also recovered for its major orders Insectivora (Eulipotyphla), Chiroptera, and Carnivora. There was no clear result from *splid* data about the relationship within Laurasiatheria, which resembles the conclusions obtained elsewhere [50, 52, 55], although results from all alignment methods support Insectivora (Eulipotyphla) as the most basal clade within Laurasiatheria [52]. The evolutionary history of bats has long been a subject of discussion, with conflicting hypothesis depending on whether morphological or molecular data was used. Earlier studies either traditionally suggested the monophyly of the suborders Megachiroptera (megabats) and Microchiroptera (microbats), e.g. [58], while other studies placed megabats together with the rhinolophoid microbats (Yinpterochiroptera), with the remaining microbats forming the suborder Yangochiroptera, e.g. [59, 60]. *Splid* data derived from most of the alignment methods support this and place *Rhinolophus ferrumequinum* as sister taxon to *Pteropus vampyrus*, while *Myotis lucifugus* was found as sister taxon to both of them. Only ProbConsRNA follows the traditional view of a monophyly of megabats and microbats and is therefore similar to the results obtained from the TBA/Multiz alignments.

The monophyly of Euarchontoglires (Euarchonta and Glires) could not be recovered from *splid* data obtained from Muscle and T-Coffee, because of the wrongly positioned Muroidea (Muscle and T-Coffee) and the wrong position of tree shrew, guinea pig, and squirrel (T-Coffee). However, all other alignment methods clearly support the monophyly of the superorder Euarchontoglires.

Among all analyzed groups, Glires are the most problematic one. Incongruent results were reported in the literature in particular concerning the position of the tree shrew. While some studies place tree shrews as sister group to Glires, others recover them as sister group to Primata (see [61] for a recent summary). *Splid* data also do not yield an unambiguous conclusion. They often place the tree shrew within (ClustalW, Mafft G-INS-i, Mafft L-INS-i) or in a sister group relationship to Glires (TBA/Multiz). Only *splid* data derived from ProbConsRNA alignments places the tree shrew (but also monophyletic Glires) within Primata. Interestingly, *splid* data obtained from the alignments of Prank recovered the tree shrew as the most basal taxon in Euarchontoglires (with monophyletic Glires as sister group to all Primates). *Splid* data from Dialign-TX, Mafft default, Muscle, and T-Coffee alignments recover Glires as polyphyletic with varying positions of the tree shrew.

Almost all methods support the monophyly of Primates, as well as a monophyly of the respective sub- and parvorders. Only *splid* data derived from the ProbConsRNA alignments places Strepsirrhini together with the tree shrew as the most basal clade within Euarchontoglires.

As a quantitatively evaluation of the mammalian tree we consider the normalized RF and quartet distances to the ENCODE reference tree, which—although not undisputed – well reflects the state of the art in mammalian phylogeny. Overall, the tree calculated from *splids* derived from the Mafft G-INS-i alignments shows the highest similary to the ENCODE reference tree (Fig. 3). The tree based on *splids* derived from the T-Coffee alignments is most different from the ENCODE tree with respect to the more sensitive quartet distance. While the tree computed with Muscle has a higher normalized RF distance, its normalized quartet distance is much lower. However, when comparing the values of the two distances for the other methods it becomes apparent that their results are quite different and show no clear correlation. For example, while the RF distances of the ENCODE tree to the trees based on the *splids* derived from the Mafft L-INS-i and Prank alignments, respectively, are similar, the quartet distances differ by a factor larger than two. Interestingly, when comparing the much more sensitive quartet distances of the trees based on *splids* extracted from the alignments calculated with ClustalW, Dialign-TX, and all three Mafft algorithms, they indicate a higher similarity to the ENCODE reference tree than the tree based on *splids* extracted from the guide tree based TBA/Multiz alignments. The Probabilistic Alignment Kit Prank [36] has been developed with a focus on a phylogenetic consistent placement of insertions and deletions. However, trees calculated from *splids* derived from Prank alignments showed no superior similarity to the ENCODE reference tree, an observation that is in line with another study [62]. We note, finally, that misplaced taxa in all trees generally had low bootstrap support.

*Data set with sequence information for at least three species.* In the following, we focus on three alignment methods to analyze *splid* performance on the large ENCODE data set: Mafft G-INS-i was chosen because it performed best on the data set containing sequence information for all taxa. In order to analyze whether the increase in the size of the data set improves the performance, we also included T-Coffee, the method with the poorest performance on the small ENCODE data set (with respect to the quartet distance). In addition, we included in our analysis the *splid* set derived from the original TBA/Multiz alignments. We removed four invariant *splid* sites extracted from the TBA/Multiz alignments, because invariant sites are not allowed when ascertainment bias correction is used.

**Table 3:** Results for the large ENCODE data set. *Splids* $\ge$ 2 bp were coded and trees were calculated with RAxML using the Gamma model for binary data and ascertainment bias correction

	Mafft G-INS-i	T-Coffee	TBA/Multiz
Number of sites	36,132,992	36,450,667	37,689,662
Number of *splids*	545,790	922,277	919,908
$d_{RF}$	16	16	12
$d'_{RF}$	0.2424	0.2424	0.1818
$d_{Q}$	5000	7494	3710
$d'_{Q}$	0.0849	0.1272	0.0630

We observed an improvement in terms of tree similarity to the ENCODE guide tree for two of the three *splid* data sets derived from the large ENCODE data set (Table 3). For the T-Coffee alignments, both tree distance measures indicate higher similarity of the maximum likelihood (ML) tree to the ENCODE guide tree ($d'_{RF} \,=\, 0.2424$ and $d'_Q \,=\, 0.1272$, respectively) than the tree calcuated from *splid* data derived from the small data set ($d'_{RF} \,=\, 0.3030$ and $d'_Q \,=\, 0.1606$, respectively). However, Boreoeutheria were not found to be monophyletic anymore: Glires are placed as sister group to ((Afrotheria, Xenarthra), Laurasiatheria) and the remaining Euarchontoglires (Primata); although with low bootstrap support. The tree shrew is recovered in a sister taxon relationship to all of the former. On the other hand, monophyly of all other major groups (Laurasiatheria, Afrotheria) and groups therein (Chiroptera, Carnivora, Insectivora, Primata etc.) was correctly recovered.

RF and quartet distance of the ML tree calculated from *splids* derived from the original TBA/Multiz alignments also decreased ($d'_{RF} \,=\, 0.1818$ and $d'_Q \,=\, 0.0630$, respectively) compared to the small ENCODE data set ($d'_{RF} \,=\, 0.2121$ and $d'_Q \,=\, 0.0668$, respectively). Monophyletic Afrotheria are recovered as sister-group to monophyletic Boreoeutheria (Epitheria) with basal Xenarthra. Within Boreoeutheria, monophyly of all major groups were correctly recovered and order within groups largely follows the ENCODE guide tree with three notable exceptions. (1) The tree shrew is now recovered as sister taxon to (Epitheria, Xenarthra). (2) Insectivora (hedgehog and shrew) is not the basal group within Laurasiatheria anymore but is now sister group to Chiroptera, (3) while both are sister group to ((Carnivora, cow), horse).

Unexpectedly, the ML tree calculated from the Mafft G-INS-i alignments (Table 3) showed a higher distance to the ENCODE guide tree tree ($d'_{RF} \,=\, 0.2424$ and $d'_Q \,=\, 0.0849$, respectively) and is thus more dissimilar than the tree calculated from *splid* data derived from the small data set ($d'_{RF} \,=\, 0.2121$ and $d'_Q \,=\, 0.0321$, respectively). Here, Laurasiatheria were not recovered to be monophyletic. Instead, non-monophyletic Insectivora are recovered as basal to ((Afrotheria, Xenarthra), Euarchontoglires) and the remaining Laurasiatheria. Again, monophyly of all other major groups (Afrotheria, Euarchontoglires) and groups therein (Primata, Glires etc.) was correctly recovered and the tree shrew was placed as sister taxon to Glires.

We note, finally, that of all species included in the large ENCODE data set, tree shrew has by far the smallest sequence coverage (approximately 10% of the amount of human sequence in the alignments), which likely contributes to its unstable position.

## Discussion

Indels are not features of individual sequences. Instead they are inferred by comparative analysis and, in practice, appear as gaps in multiple sequence alignments. In some alignment methods they are explicitly modelled and contribute to the score, e.g. by means of affine gap costs. In other approaches they are modelled only implicitly. It is not unexpected, therefore, that the number and position of gaps depends quite strongly on the alignment algorithm. The fact that the choice of the alignment algorithm has an impact on the reconstructed phylogenies is well documented in the literature, see e.g. [63–67]. Nevertheless, gap positions can be phylogenetically informative.

We have focused here on a subclass of indels, namely those which can be found in more than one sequence and therefore define a split in the taxon set. Our definition and inference of such split-inducing indels (*splids*) is based on two basic principles that are largely accepted in the literature. First, indels at the same position, i.e. sharing the same end points in two sequences, are likely homologous. Second, independent single-residue insertions and deletions tend to occur more frequently than multi-residue indels. Hence they are expected to contribute a more noisy signal and hence are disregarded in our analysis.

We have tested the information content of *splids* on three simulated and two real-life data sets and analyzed the capability of *splids* introduced by nine different alignment programs for phylogenetic inference by ML. For artificial data sets, which are generated from a known underlying phylogeny, we find that *splid*-based ML reconstruction leads to nearly perfect trees. On the real-life data sets, however, we observe larger discrepancies between different alignment methods.

The *splid*-based phylogenies clearly recovered most of the undisputed monophyletic groups. Although there are clear differences in the alignment methods, the approach is surprisingly robust across a wide variety of alignment techniques. We expected a large influence of the guide tree on the reconstructed phylogeny since guide trees are known to influence the indel pattern [68]. Nevertheless, we observed that this effect is small for indel-based phylogenies when only *splids* are considered. Overall, alignment methods that put more emphasis on modelling indels, in particular those that employ an affine gap cost model, perform superior to alignment algorithm that consider indels only implicitly. Furthermore, for very large data sets we can observe a decreasing influence of the alignment algorithm.

Similar to other phylogenetic approaches, taxon sampling has a major influence on branch positions in very divergent taxonomic orders. This can been seen for example in the Laurasiatheria, where a small group of more closely related taxa (e.g. bats or Carnivora) is embedded in a larger set of more distantly ones. While *splid* data always supports a monophyly of Chiroptera, their position within Laurasiatheria cannot be unambiguously determined.

Increasing sequence length, and therefore *splid* information, does not necessarily lead to better resolved trees. This effect is likely related to the observation that alignments computed for large data sets have relatively large error rates, especially when sequence coverage is low. This in turn introduces considerable conflicting signal in tree inference [69]. In the case of low but roughly equal amount of sequence data for all taxa, the choice of the alignment algorithm seems to have a higher effect within lower taxonomic orders, while groups resembling higher taxonomic orders are relatively stable and are mostly correct positioned.^3^

## Additional file

**Additional file 1: Figure S1.** Normalized quartet distances for the simulated data sets.

## Footnotes

1 http://hgdownload.cse.ucsc.edu.

2 http://hgdownload.cse.ucsc.edu/goldenPath/hg18/encode/MSA/DEC-2007/.

3 http://tu-dresden.de/zih/.
