# Supplementary material for: Split-inducing indels in phylogenomic analysis
Source: Algorithms Mol Biol. 2018 Jul 16;13:12. doi: 10.1186/s13015-018-0130-7 (PMC6047143; doi:10.1186/s13015-018-0130-7)
Supplement: Supplementary file 1 — Additional file 1: Figure S1. Normalized quartet distances for the simulated data sets. [file 13015_2018_130_MOESM1_ESM.pdf]

Additional file 1

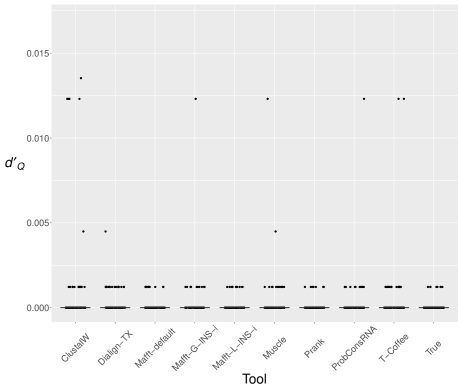

(a) Geometric distribution with $q = 0.55$.

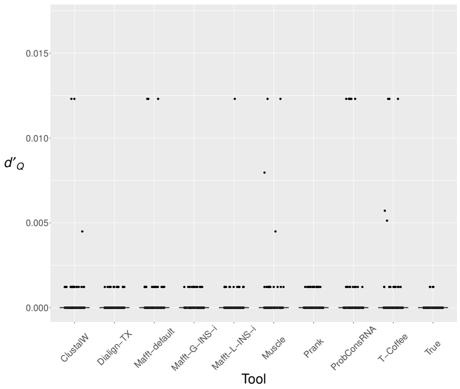

(b) Geometric distribution with $q = 0.7$.

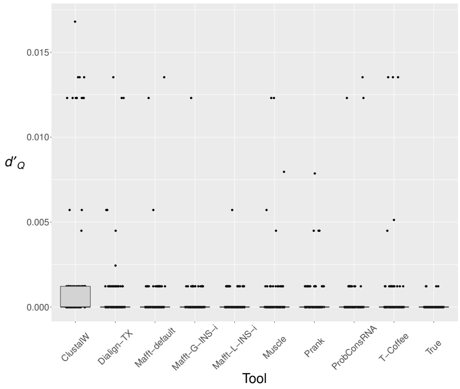

(c) Lavalette distribution.

Figure S1: Normalized quartet distances (d'_Q) for the data sets simulated with three different indel models and realigned with various methods. True = Scores obtained from trees calculated from the 'true' `INDELible` alignments. See main text for further details.
